# Medical and Philosophical News

**Published:** 1781-02

**Authors:** 


On the 9th of December 1780, the College of Physicians at Paris held their Anniversary Meeting in the schools of the Sorbonne. On this occasion were delivered the Eulogies of Doctors Joseph de Jussieu, Albert Hazon, and William Michel, three members of the college, who died in the course of the preceding year. M. Destandes read a dissertation condemning the use of opium in intermittents, previous to the paroxysm, a practice introduced about thirty years ago by M. Berryat, physician at Auxerre. M. Mallet gave an account of a new species of bark brought from Martinico, and which seems to be the same as that lately described in the *Phil. Transf.* by Dr. Wright. M. Majault communicated some observations on vinegar, proving it not to be an antidote to arsenic, as some have lately asserted.

